# Growth-inhibition of cell lines derived from B cell lymphomas through antagonism of serotonin receptor signaling

**DOI:** 10.1038/s41598-019-40825-x

**Published:** 2019-03-12

**Authors:** Shrikant S. Kolan, Tommy Lidström, Tomás Mediavilla, Andy Dernstedt, Sofie Degerman, Magnus Hultdin, Karl Björk, Daniel Marcellino, Mattias N. E. Forsell

**Affiliations:** 1 Department of Clinical Microbiology, Section of Infection and Immunology, Umeå University, Umeå, Sweden; 2 Department of Integrative Medical Biology, Umeå University, Umeå, Sweden; 3 Department of Medical Biosciences, Umeå University, Umeå, Sweden

## Abstract

A majority of lymphomas are derived from B cells and novel treatments are required to treat refractory disease. Neurotransmitters such as serotonin and dopamine influence activation of B cells and the effects of a selective serotonin 1A receptor (5HT1A) antagonist on growth of a number of B cell-derived lymphoma cell lines were investigated. We confirmed the expression of 5HT1A in human lymphoma tissue and in several well-defined experimental cell lines. We discovered that the pharmacological inhibition of 5HT1A led to the reduced proliferation of B cell-derived lymphoma cell lines together with DNA damage, ROS-independent caspase activation and apoptosis in a large fraction of cells. Residual live cells were found ‘locked’ in a non-proliferative state in which a selective transcriptional and translational shutdown of genes important for cell proliferation and metabolism occurred (*e*.*g*., AKT, GSK-3β, cMYC and p53). Strikingly, inhibition of 5HT1A regulated mitochondrial activity through a rapid reduction of mitochondrial membrane potential and reducing dehydrogenase activity. Collectively, our data suggest 5HT1A antagonism as a novel adjuvant to established cancer treatment regimens to further inhibit lymphoma growth.

## Introduction

B cell lymphomas represent a heterogeneous group of genetically, phenotypically and clinically distinct malignancies. Frequently, the oncogenic transformation of B cells occurs during the germinal center reaction and gives rise to a number of lymphoma types, including diffuse large B cell lymphomas (DLBCL), Burkitt lymphomas (BL) or follicular lymphomas^1–3^. Both DLBCL and BL have very high proliferation indices in which prompt diagnosis followed by an immediate start of therapy is critical for a positive clinical outcome^4^. Standard chemotherapy in combination with anti-CD20 antibody treatment is frequently used for DLBCL patients, and BL is treated with multiagent chemotherapy regimens with or without anti-CD20 antibody treatment^5–7^. In addition, B cell lymphomas remain a significant cause of morbidity in patients with background infections, such as HIV-1^5,8^. In patients with relapse or refractory disease, salvage chemotherapy is often insufficient and results in substantially worse prognosis. New modalities or improvements to current treatment regimens for B cell lymphomas are therefore warranted^9^.

The role of the monoamine serotonin as a neurotransmitter within the central nervous system is well established. Serotonin signaling in the brain regulates many vital processes, such as sleep, appetite and mood^10^. However, the majority of serotonin in the body is found peripherally, where it is produced by enterochromaffin cells in the gut and carried by platelets in circulation^11,12^. Recently, there has been an increased interest in how neurotransmitters regulate cells of the adaptive immune system. For example, it has been shown that activated splenic T cells have the capability to produce both serotonin and dopamine^13,14^. A number of serotonin receptors and dopamine receptors are expressed by B cells and have been reported to play an important role in activation and proliferation of these, *in vitro*^15,16^. Collectively, this indicates a potential role of neurotransmitters for direct regulation of T cell-dependent B cell activation in the germinal center^15^. If germinal center-derived B cell lymphomas retain expression of serotonin and/or dopamine receptors, it is possible that well-defined neuropharmacological drugs may be repositioned and used as an auxiliary therapy to currently used treatment regimens for a range of B cell-malignancies^15^.

Here, we investigate the potential of a selective serotonin receptor (5HT1A) antagonist to influence the proliferation and survival of B cell-derived lymphomas, and characterize key cellular and molecular pathways affected by treatment.

## Materials and Methods

An extended version of methods section is available in the Supplemental data sheet.

### Ethics and patient samples

Patient samples were acquired from the University Hospital of Northern Sweden, Umeå. The study was approved by Uppsala ethics board (Dnr 2014/233) and informed consent from all participants was obtained according to the Declaration of Helsinki. Patient material included lymph node or spleen biopsies of 10 patients aged 18–84 years that had been diagnosed with DLBCL (N = 4), chronic lymphocytic lymphoma (CLL, N = 2), follicular lymphoma grade 1–2 (N = 2). Biopsies from lymph nodes were used as control (N = 2). Diagnosis was based on morphology, flow cytometry immunophenotyping and immunohistochemistry. In all tumor samples >50% malignant cells were found.

### Cell lines and reagents

Human B-cell derived lymphoma cell lines of BL (Raji, Dg-75, Rael, Ramos, and Bjab), DLBCL (Val, Opl-2), chronic lymphocytic leukemia (Mec-1, Mec-2) and Mantle cell lymphoma (MCL, Granta) were kindly provided by George and Eva Klein, MTC, Karolinska institutet^17–25^. All cell lines were maintained in either RPMI-1640 or IMDM medium (Thermo Fischer) supplemented with 10% heat inactivated fetal bovine serum (FBS) and 1% penicillin–streptomycin (Gibco) at 37° with 5% CO_2_. 5HT1A antagonist WAY 100,635 (#W108, hereafter as WAY) was purchased from Sigma-Aldrich.

### Cell proliferation, BrdU incorporation and microscopy

Cells were subjected to serum starvation for 1 h in serum-free media before addition of the selective 5HT1A antagonist WAY and/or 10%FBS. Cell counting was performed with trypan blue staining and a TC20 automated cell counter (Bio-Rad) or by flow cytometric analysis using fixed amount of beads, at indicated time points. For BrdU incorporation experiments, Bjab cells were pulsed with 10 µM Bromodeoxyuridine (BrdU) (Life Technologies) for 4 h prior to harvest and percentage of BrdU positive cells were quantified using a LSRII (BD) flow cytometer with FlowJo v10.0.7 (FlowJo, LLC) software. At 72 h, control and WAY treated (50 µM/24 hr) Bjab cells were subjected to phase contrast microscopy and images were taken using EVOS FL Cell Imaging System (Thermo Fischer) with 20× magnification; (scale bar 50 µm).

### RNA extraction and Quantitative real time PCR (rtqPCR)

Total cellular RNA was extracted using either Trizol (Invitrogen) or RNAeasy mini kit (Qiagen) according to manufacturer’s instructions. Reverse transcription was performed using High capacity RNA-to-cDNA kit according to manufacturer’s instructions (Applied Biosystems). Expression levels of *HTR1A*, *MYC*, *TP53*, *Gsk3β*, *Akt1*, *Bcl2*, *Bcl2l11 (BIM)*, *beta actin* and *18* *s rRNA* were measured by rtqPCR and by using TaqMan Gene Expression Assays-on-Demand (Applied Biosystems). rtqPCR reactions were performed in triplicate with ABI prism 7900 HT sequence detection system or Quant Studio 5 (Applied Biosystems). Expression levels were normalized to reference gene and were analyzed by using 2^(−ΔΔct)^ method as described by Livak and Schmittgan^26^. First, the level of target gene was normalized to reference gene by calculating ΔCt value [ΔCt = _target gene_ − Ct _reference gene_] formula. Thereafter, ΔΔCt was calculated based on [ΔCt _target_ − ΔCt _calibrator/ control_] formula, while fold change difference was determined by evaluating the expression 2^(−ΔΔct)^.

### Cell-cycle, apoptosis and ds-DNA damage analysis

Cell cycle analysis was performed according to Vindelov *et al*.^27^. At 72 h, control and WAY treated Bjab cells were harvested and washed twice with PBS. Next, cell nuclei were isolated and incubated with propidium iodide (PI) in darkness for at least 30 minutes at 4 °C. Nucleic content of cells and cell-cycle distribution were analyzed by flow cytometry. Analyses for cell apoptosis and ds-DNA damage were conducted by using Annexin-V FITC/PI apoptosis detection kit or Apoptosis, DNA damage and cell proliferation kit (BD Biosciences) respectively, as per manufacturer protocols. Apoptotic cells were defined as annexin V^+^/PI^+^ population. In ds-DNA damage experiments, control and WAY treated Bjab cells were stained with phosphorylated H2AX (ps139) and cleaved PARP (Asp214). All samples were analyzed using LSRII (BD) flow cytometer with FlowJo v10.0.7 (FlowJo, LLC) software.

### Dehydrogenase activity determination (WST-8 assay)

Cellular dehydrogenase activity was determined using Cell Counting Kit-8 (CCK-8 Dojindo Laboratories). In brief, Bjab cells (1 × 10^5^ cells/well) were seeded in 96-well flat-bottom plate for 24 h and exposed to either single treatment (10/25/50/75/150 µM) or repetitive treatment (50 µM/24 h) of WAY for next 24 h or 72 h respectively. At the end of incubation period 10 µl of CCK-8 solution was added to each well and post 4 h incubation absorbance was measured on a microplate reader (Varioskan Flash, Thermo Fischer) at 450 nm.

### Measurement of Reactive oxygen species (ROS) and mitochondrial membrane potential (∆Ψm)

Intracellular ROS production was assessed by using a 2′,7′-dichlorodihydrofluorescein diacetate dye (H_2_DCFDA-Thermo Fischer) as per manufactures protocol. Mitochondrial membrane potential (∆Ψm) was determined by using JC-1 staining following the manufacturer protocol (JC-1 Mitochondrial Membrane Potential Detection Kit, Thermo Fischer). All samples were analyzed by either using Accuri or LSRII (BD) flow cytometer and data was analyzed using FlowJo v10.0.7 (FlowJo, LLC) software.

### Protein expression

Qualitative or quantitative measurements of translation was performed by western blot using the following antibodies: cMYC, p53, pAKT-s473, total AKT, pGSK3-β(s9), total GSK3-β, Beta-actin (all from Cell Signaling), pH2AX-ser139 (Millipore), and 5HT1A (Santa Cruz Biotechnology), and Cyclophilin-A (Thermo Fischer). Antibody binding was detected with Supersignal West Pico Chemiluminescent substrate (Thermo Fischer) and visualized using ImageQuant LAS4000 (GE Healthcare). Intensity of bands was quantified using image analysis software from Licor (Lincoln).

### Detection of autophagy

Details of preparation of mCherry-EGFP-hLC3 retroviral particles and generation of the mCherry-EGFP-hLC3 reporter cell line (Bjab-mCGhLC3) are provided in online Supplemental data sheet. For the quantification of autophagy, control and WAY treated mCherry-EGFP-LC3B-expressing Bjab cells were subjected to flow cytometry [BD FACSAria™ III (BD Biosciences)] and data was analyzed using FlowJo v10.0.7 (FlowJo, LLC) software.

### Statistical analysis

Statistical analysis were performed with GraphPad Prism 7.0 (GraphPad software) and appropriate test, as indicated.

## Results

### Primary B cell-derived lymphomas and lymphoma-derived cell lines represent potential targets for modulation of 5HT1A receptor signaling

**Figure 1:**
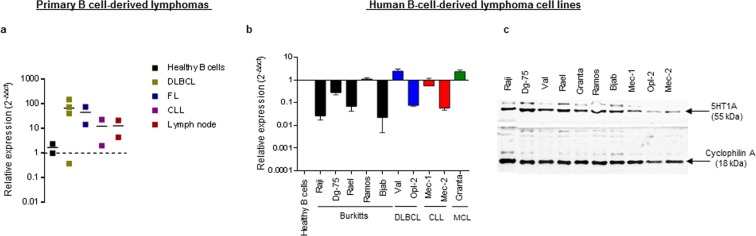
Primary B cell-derived lymphomas and lymphoma-derived cell lines express 5HT1A. (**a**) rtqPCR analysis of 5HT1A expression in lymph node or spleen biopsies samples from different types of primary NHLs. The results are presented as a fold change relative to B cells from healthy donors, expression levels were normalized to of Beta-actin and calculated by 2(−ΔΔct) method. (**b**) rtqPCR analysis of 5HT1A expression in different types of B cell-derived lymphoma cell lines. The results are presented as a fold change relative to B cells from healthy donors, expression levels were normalized to 18 s rRNA and calculated by 2(−ΔΔct) method. Data is presented as the mean of 2 independent experiments ± SD. (**c**) Western blot analysis of 5HT1A protein expression in B cell-derived lymphoma cell lines (n = 3). The gel and blot image shown in the figure was cropped from same part of the gel with an exposure time of 30 s where Cyclophilin-A was used as a loading control. Full-length blots and gels are presented in Supplementary Figure 3.

Transcriptional and translational expression of 5HT1A has previously been demonstrated in murine B cells^28^. By rtqPCR, we could detect mRNA encoding 5HT1A in purified human B cells, in biopsies from malignant lymph nodes from clinically diagnosed lymphoma patients, and in control biopsies taken from healthy lymph node tissue (Fig. 1a). Similarly, using rtqPCR and western blot analysis, we demonstrated 5HT1A mRNA (Fig. 1b) and protein expression (Fig. 1c and Supplementary Fig. S1) in cell lines derived from BL, DLBCL, chronic lymphocytic leukemia and mantle cell lymphoma. These data establish that human B cells, tissue samples from primary B cell-derived lymphoma and B cell-derived lymphoma cell lines express 5HT1A at both transcriptional and translational level.

### A selective 5HT1A antagonist can irreversibly inhibit proliferation of a BL cell-line in a concentration-dependent manner

**Figure 2:**
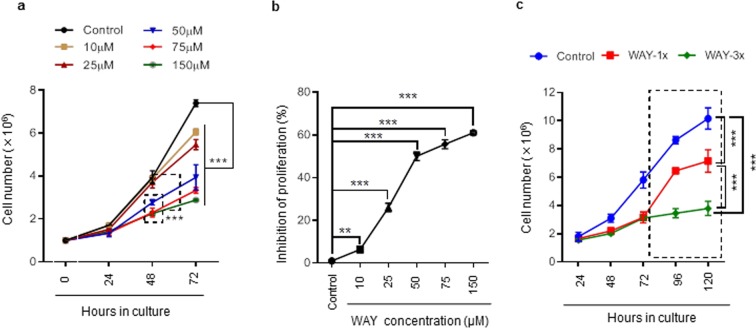
The 5HT1A antagonist WAY inhibits the proliferation of Bjab (BL) cell lines. (**a**) Bjab cells were treated once with various concentration of WAY for indicated periods and cells were counted every 24 h by using TC20 Automated cell counter. Data is presented as the mean of 4 independent experiments ± SD. Statistical comparison of control and treated cells at 48 and 72 h is indicated (^***^P < 0.001, Two-way ANOVA test). (**b**) Dose response curve displaying inhibition of proliferation in Bjab cells at 72 h. Data is presented as the mean of 3 independent experiments ± SD. Statistical comparison of control and treated cells is indicated (^**^P < 0.01; ^***^P < 0.001; Two-way ANOVA test). (**c**) Bjab cells were exposed to single (WAY-1×; 50 µM) and repetitive treatments (WAY-3×; 50 µM/24 h) for 72 h. At 72 h cell culture media was replaced with fresh cell culture media (indicated as a dotted square) devoid of antagonist and numbers of cells were counted for the next 48 h (i.e. for 96 and 120 h). Data is presented as the mean of 3 independent experiments ± SD. Statistical comparison of control and treated cells at 72 h is indicated (^***^P < 0.001; Two-way ANOVA test).

To address whether a blockade of serotonin signaling could inhibit proliferation of B cell-derived lymphomas, we treated the BL cell line Bjab with increasing concentrations of WAY and subsequently quantified the number of cells in culture over a 72 h period. Treatment did not have a major impact after 24 h, but resulted in a significant reduction in total cell numbers at 48 and 72 h (Fig. 2a). Importantly, WAY inhibited Bjab cell proliferation in a concentration-dependent manner in which the maximum inhibition was approximately 60% and required between 75 µM and 150 µM of WAY (Fig. 2b). To better understand the functional and molecular consequences of 5HT1A antagonism on Bjab cells, we chose to further evaluate 50 µM for subsequent experiments. At this concentration, approximately 50% inhibition had been achieved at the 72 h time point (Fig. 2b). Due to 5HT1A recycling^29^ and/or metabolism of the antagonist^30^, and the potential of WAY to induce receptor desensitization by internalization^31^, we compared the inhibitory effects after a single treatment of 50 µM WAY with treatment of 50 µM WAY every 24 h. Quantitative cell counting demonstrated that both treatment regimens inhibited cell proliferation to a similar degree after 48 and 72 h in culture (Fig. 2c).

To determine whether the inhibition of cell proliferation was reversible, cells were washed after 72 h and supplemented with fresh medium devoid of antagonist for an additional 48 h. Strikingly, Bjab cells that had been exposed to a single treatment with WAY recovered in total cell numbers over the subsequent 48 h period whereas Bjab cells that had been subjected repetitive treatment regimen did not (Fig. 2c). In conclusion, repeated treatment with a selective 5HT1A antagonist leads to an irreversible inhibitory effect for proliferation of Bjab cells.

### Repetitive treatment with WAY induces DNA-damage, ROS-independent caspase-activation and apoptosis

**Figure 3:**
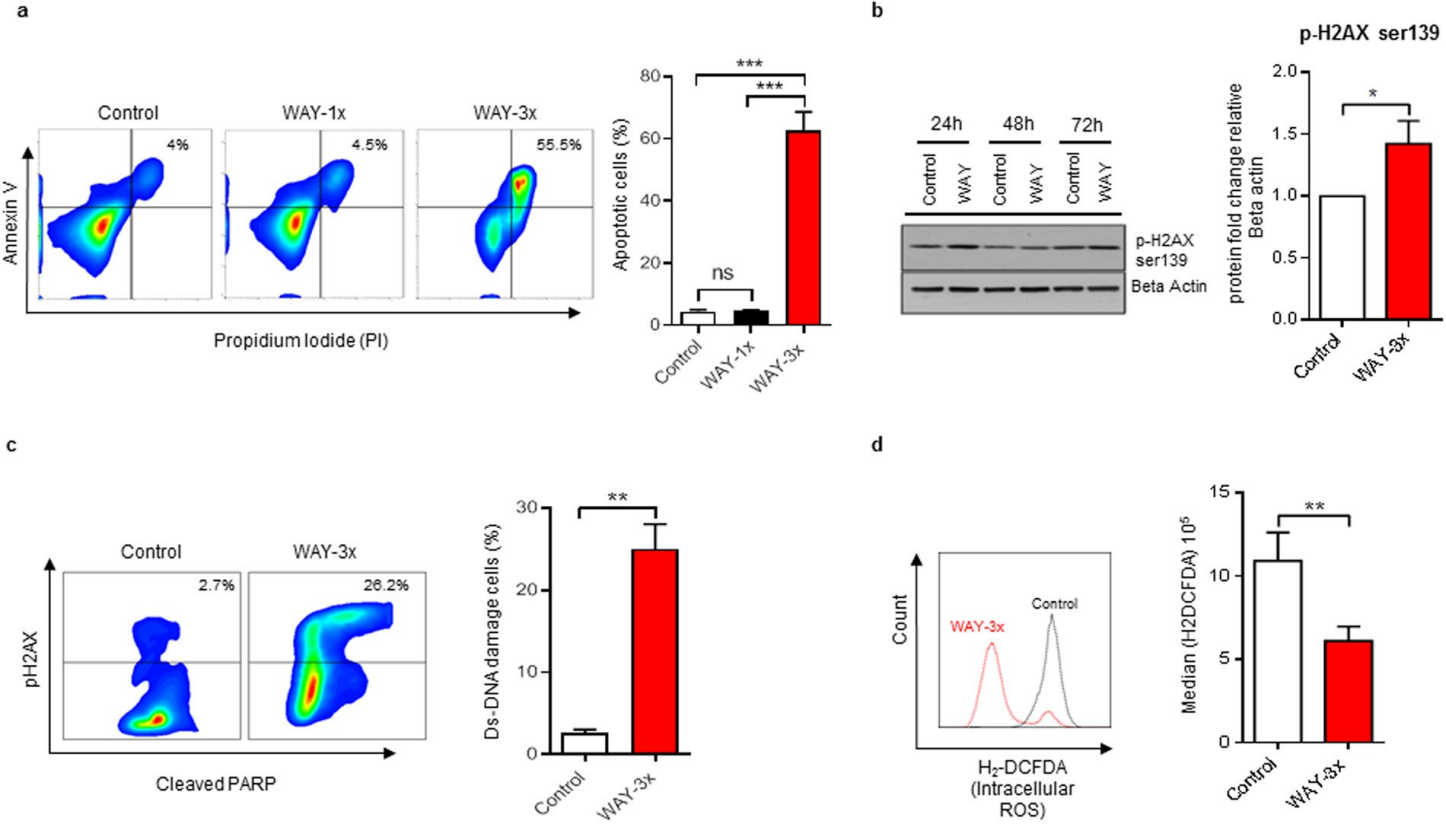
Repetitive treatments of the 5HT1A antagonist WAY induces apoptosis, DNA damage and oxidative stress in Bjab cells. (**a**) Bjab cells were exposed to single (WAY-1×; 50 µM) and repetitive treatments (WAY-3 × ; 50 µM/24 h) for 72 h, stained with annexin-V and PI, and analyzed by flow cytometry. Data is presented as the mean of 3 independent experiments ± SD. Statistical comparison of control and treated cells is indicated (ns: non-significant; ^***^P < 0.001; Two-way ANOVA test). (**b**) Cellular levels of pH2AX-ser 139 were detected by Western blot using Beta-actin as a loading control. Band intensity was quantified using image analysis software from Li-cor and illustrated in the bar diagram. Data is presented as the mean of 4 independent experiments ± SD. Statistical comparison of control and treated cells is indicated (^*^P < 0.05; Mann-Whitney test). The blot image shown in the figure was cropped from same part of the gel with an exposure time of 60 sec. Clear delineation with white line is used. Full-length blots and gels are presented in Supplementary Figure 4. (**c**) Bjab cells were exposed to repetitive treatments (WAY-3×; 50 µM/24 h) for 72 h, stained with anti-pH2AX-ser 139 and anti-cleaved PARP, and analyzed by flow cytometry. Data is presented as the mean of 3 independent experiments ± SD. Statistical comparison of control and treated cells is indicated (^**^P < 0.01; Mann-Whitney test). (**d**) Histogram depicting intracellular ROS response as measured with carboxy-2′,7′-dichlorodihydrofluorescein diacetate H2DCFDA by flow cytometry in control and WAY (WAY-3×; 50 µM/24 h) treated Bjab cells for 72 h. Data is presented as the mean of 6 independent experiments ± SD. Statistical comparison of control and treated cells is indicated (^**^P < 0.01; Mann-Whitney test).

Flow cytometric analysis using propidium idodide/Annexin V staining revealed that approximately 62% of all cells after repetitive treatment adapted a state of apoptosis (PI^+^/AxV^+^) at 72 h. At the same time point, only an average of 4.3% or 4.5% of cells were apoptotic in control cells or cells treated only once, respectively (Fig. 3a). Consistent with apoptosis, a significant number of cells (average 31%) in the repetitively treated group had disrupted membrane integrity, as measured by a fixable viability dye (Supplementary Fig. S2).

Phosphorylation of the H2A histone family member X (pH2AX) is a cellular response to double-strand DNA breaks^32^. By western blot, we observed an increased level of pH2AX (Ser139) in repetitively treated Bjab cells as compared to control cells at 72 h (Fig. 3b and Supplementary Fig. S3). Flow cytometric analysis also confirmed a significant increase in cleaved poly (ADP-ribose) polymerase (PARP) (Fig. 3c) which suggested that the treatment regimen had induced caspase activation^33^. Excessive ROS-production due to cellular stress may lead to caspase activation and apoptosis^34^. However, repetitively treated cells had significantly reduced levels of intracellular ROS in comparison to control cells (Fig. 3d). Collectively, these data suggested that repetitive treatment of Bjab cells with WAY induced DNA-damage, ROS-independent caspase-activation and apoptosis in a significant number of cells.

### Cell cycle arrest and induction of polyploidy by inhibition of 5HT1A in Bjab cells

**Figure 4:**
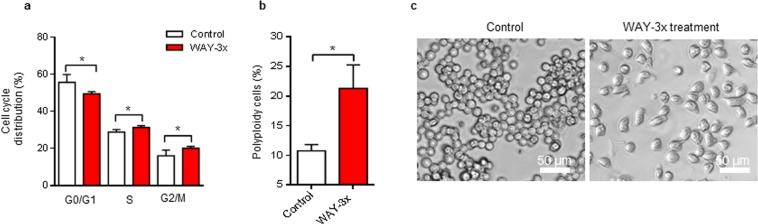
Repetitive treatments of the 5HT1A antagonist WAY induces cell-cycle arrest, polyploidy and morphological alterations in Bjab cells. (**a**,**b**) Cell-cycle analysis of Bjab cells exposed to repetitive treatments (WAY-3×; 50 µM/24 h) for 72 h. Cells were stained with PI and analyzed for cell-cycle distribution (**a**) and polyploidy cells (**b**) by flow cytometry. Data is presented as the mean of 4 independent experiments ± SD. Statistical comparison of control and treated cells is indicated (^*^P < 0.05; Mann-Whitney test). (**c**) Observation of cellular morphology upon repetitive treatments (WAY-3×; 50 µM/24 h) for 72 h. Images were captured in EVOS FL Cell Imaging System (Thermo Fischer) using phase contrast microscopy (20× magnification; scale bar 50 µm). Representative images of control and WAY treated Bjab cells are shown.

Since up to 40% of Bjab cells remained viable following treatment, but were unable to proliferate after removal of the antagonist (Fig. 2c), we performed cell-cycle analysis to investigate if cells were ‘locked’ in a specific phase of the cell cycle. Flow cytometric analysis revealed a minor increase of cells in the S and G2/M phases and a minor decrease of cells in G0/G1 phase (Fig. 4a). Interestingly, while we did not find a major accumulation of cells in a specific stage of the cell cycle between the groups, the repetitive treatment had induced significant polyploidy in Bjab cells (Fig. 4b). This was associated with a treatment-induced morphological change at 72 h, where untreated Bjab cells maintained their rounded shape while repetitively treated Bjab cells appeared to increase in size had acquired an elongated shape (Fig. 4c).

Collectively, these data suggested that 5HT1A antagonist WAY had induced aberrant cell cycle progression and a morphological change in a significant population of Bjab cells after repetitive treatment.

### Reduced BrdU incorporation and selective down-regulation of cell cycle and metabolic related genes

**Figure 5:**
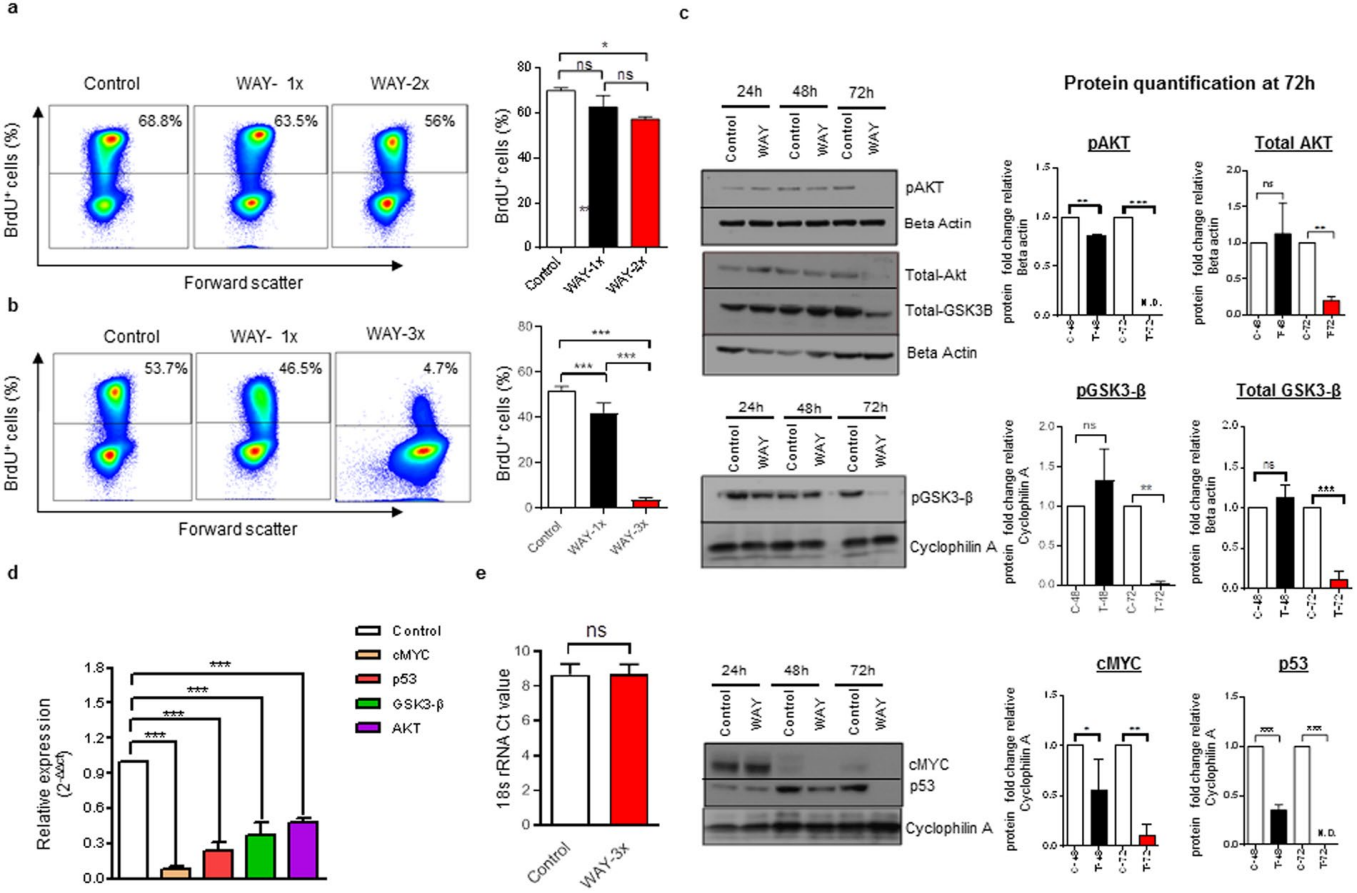
Repetitive treatments of the 5HT1A antagonist WAY results in reduced DNA synthesis and down-regulation of cell cycle and metabolic genes. (**a**,**b**) Representative flow cytometric analysis plots for BrdU+ cells at 48 and 72 h respectively. Bjab cells were exposed to indicated treatments of WAY (**a**) 1× (50 µM) or 2× (50 µM/24 h) for 48 h or (**b**) 1× (50 µM) or 3× (50 µM/24 h) for 72 h. In the last 4 h of incubation, cells were pulsed with BrdU (10 µM) and subjected to flow cytometric analysis. Data is presented as the mean of 3 independent experiments ± SD. Statistical comparison of control and treated cells is indicated (^*^P < 0.05; ^***^P < 0.001; Two-way ANOVA test). (**c**) Bjab cells were exposed to repetitive treatments (WAY-3×; 50 µM/24 h) for 72 h and protein expression of pAKT, Total AKT, pGSK3-β, Total GSK3-β, cMYC and p53 were detected by Western blot using Beta-actin or Cyclophilin-A as loading controls. Bands were quantified using image analysis software from Li-cor and illustrated in the bar diagram. Data is presented as the mean of 4 independent experiments ± SD. Statistical comparison of control and treated cells is indicated (ns: non-significant; ^*^P < 0.05; ^**^P < 0.01; ^***^P < 0.001; Two-way ANOVA test). The blot images shown in the figure were cropped from same part of the gel with an exposure time of 60 sec. Clear delineation with white line is used. Full-length blots and gels are presented in extended Supplementary Figure 5. (**d**) rtqPCR analysis of cMYC, p53, GSK3-β and AKT1 expression in Bjab cells exposed to repetitive treatments (WAY-3×; 50 µM/24 h) for 72 h. Expression levels were normalized to 18 s rRNA, calculated by 2(−ΔΔct) method and presented as a fold change compared to control cells. Data is presented as the mean of 3–4 independent experiments ± SD (^***^P < 0.001; Two-way ANOVA test). (**e**) rtqPCR analysis for 18 s rRNA, expression in Bjab cells exposed to repetitive treatments (WAY-3×; 50 µM/24 h) and control cells at 72 h (n = 6). Statistical comparison of control and treated cells is indicated (ns: non-significant; Mann-Whitney test).

In order to understand why remaining viable Bjab cells could not divide after removal of antagonist, we determined the potential of these cells to generate new genomic DNA by pulsing cells with BrdU after 48 or 72 h of treatment. Consistent with the relatively similar growth rate between single and repetitively treated Bjab cells (Fig. 2c), the capacity to generate new genomic DNA between untreated, single treated and repeatedly treated Bjab cells was relatively similar at the 48 h time point (average untreated: 70.2%; single treated: 67.6%; repetitively treated: 58.2% BrdU^+^ cells, Fig. 5a). In contrast, repeatedly treated Bjab cells had lost the capacity to generate new genomic DNA, in comparison with the untreated or single treated group at the 72 h time point (average untreated: 51.8%; single treated: 42.1%; repetitively treated 3.7% BrdU^+^ cells, Fig. 5b). This suggested that repetitive treatment with the 5HT1A antagonist WAY had induced senescence in viable and non-apoptotic Bjab cells.

Protein kinase B (AKT) is a major intracellular signaling kinase regulating a number of cellular events including cell cycle progression, proliferation, nutrient metabolism and cell survival^35^. Since 5HT1A signaling has been shown to regulate AKT^36^, we subjected Bjab cells to repeated treatments of WAY and analyzed total-and phosphorylated levels of AKT (p-AKT) by western blot. Consistent with a role of 5HT1A in the regulation of AKT, we found a significant reduction in total AKT at 72 h (Fig. 5c and Supplementary Fig. S4**)**. However, at 48 h both treated and untreated groups had similar levels of total AKT. In contrast, the 5HT1A antagonist treatment had induced a significant reduction in p-AKT at 48 h that was further reduced to undetectable levels at 72 h (Fig. 5c and Supplementary Fig. S4**)**. The treatment-induced reduction of total AKT protein at the 72 h time point was corroborated by a significant reduction of mRNA transcripts for AKT at 72 h (Fig. 5d). This suggested that AKT signaling was affected both on transcriptional, translational and post-translational level.

To further investigate the impact of the 5HT1A antagonist on Bjab cells, we assessed both the transcriptional and translational expression of a number of genes important for oncogenic transformation of B cells. Glycogen synthase kinase 3 beta (GSK3-β) is a serine/threonine kinase and identified as a target for AKT^37^. We found that translation of total GSK3-β and phosphorylated GSK3-β was unaltered at 48 h but that both were majorly reduced after 72 h of repeated treatment with the 5HT1A antagonist (Fig. 5c and Supplementary Fig. S4). In addition, translation of p53 was reduced at 48 h and was low or undetectable at 72 h, and while cMyc is naturally reduced in Bjab cells during cell culture^38,39^, WAY treatment led to an additional and rapid downregulation of cMyc (Fig. 5c and Supplementary Fig. S4). This reduction was also reflected in a significant decrease of mRNA transcripts for GSK3-β, cMyc and p53 in treated cells (Fig. 5d).

In the same setting, we could confirm that transcripts for 18 s rRNA was similar between treated and untreated groups (Fig. 5e). This suggested that WAY had induced transcriptional repression of specific signaling pathways related to cell cycle progression and metabolism, and of transcripts for mutated p53 in Bjab cells^40^.

### WAY treatment results in reduced dehydrogenase activity, mitochondrial membrane potential and increased autophagic flux

**Figure 6:**
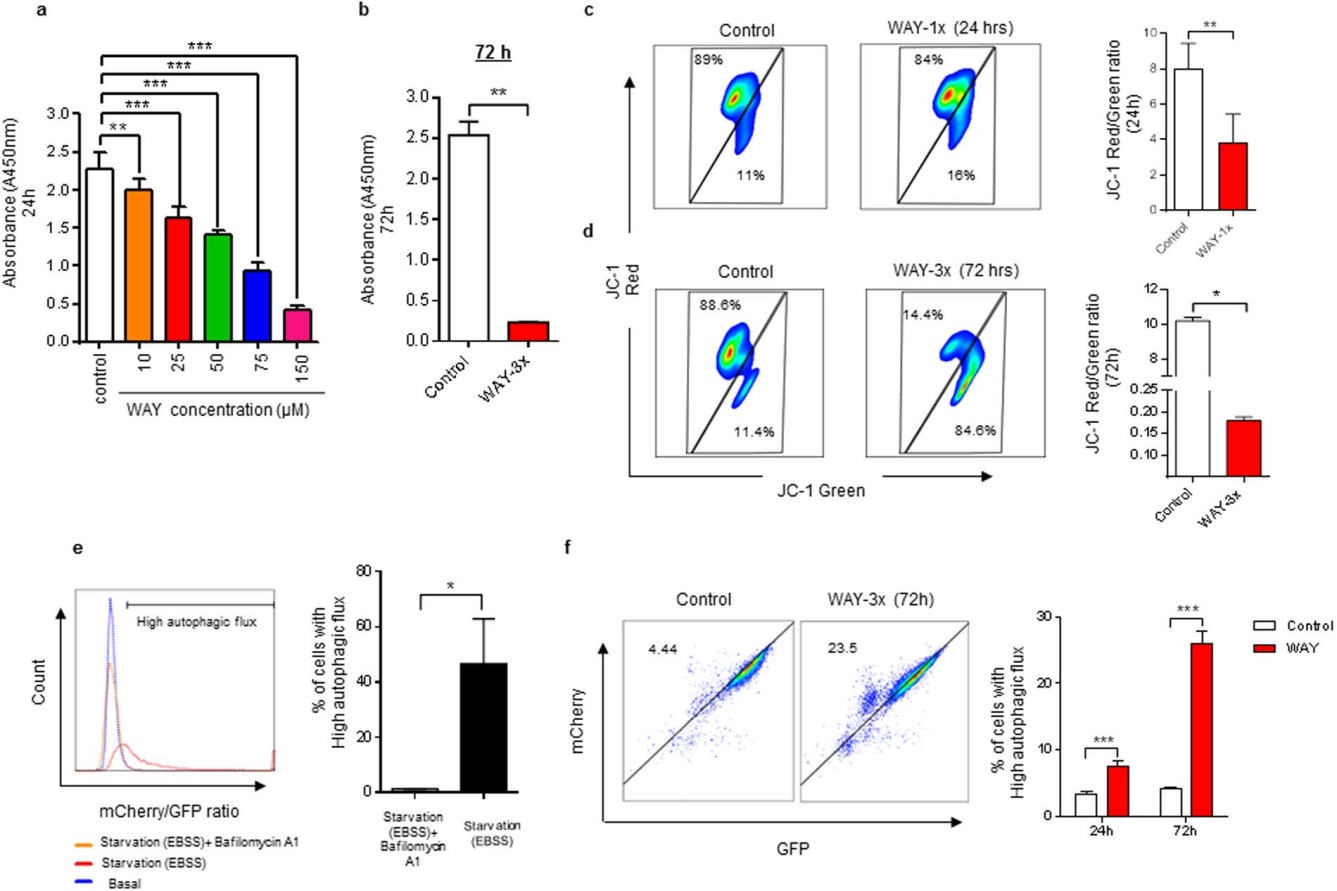
5HT1A antagonist WAY treatment results in reduced mitochondrial membrane potential, dehydrogenase activity with concomitant increase autophagic flux. (**a**,**b**) Bjab cells were exposed to, increasing concentration of WAY for 24 h (**a**) or repetitive treatments (WAY-3×; 50 µM/24 h) for 72 h (**b**) and cells were analyzed for cellular dehydrogenase activity by using CCK-8 assay kit. Data is presented as the mean of 3 independent experiment ± SD. Statistical comparison of control and treated cells is indicated (^**^P < 0.01; ^***^P < 0.001; Two-way ANOVA test). (**c,d**) Representative flow cytometric analysis plot of mitochondrial membrane potential (∆Ψm) determination at 24 and 72 h respectively. Bjab cells were exposed to single treatment (WAY-1×; 50 µM) or repetitive treatment (WAY-3×; 50 µM/24 h) for 24 or 72 h respectively and analyzed for ∆Ψm by flow cytometry. Data is presented as the mean of 3 independent experiment ± SD. Statistical comparison of control and treated cells is indicated (^*^P < 0.05; ^**^P < 0.01; Mann-Whitney test). (**e**) Histogram illustrating the quantification of autophagy flux. mCherry-EGFP-LC3B-expressing Bjab cells were treated with Earle’s Balanced Salt Solution (EBSS) (starvation is the most potent autophagy stimulus) and EBSS in the presence of Bafilomycin A1 (autophagy inhibitor) and compared to basal autophagy rate. Data is presented as the mean of 3 independent experiment ± SD. Statistical comparison of Bjab cells treated with EBSS and EBSS in the presence of Bafilomycin A1 is indicated (^*^P < 0.05; Mann-Whitney test). (**f**) mCherry-EGFP-LC3B-expressing Bjab cells were exposed to single treatment (WAY-1×) for 24 h or repetitive treatments (WAY-3×; 50 µM/24 h) for 72 h and autophagic flux was measured by flow cytometry. Cells with high autophagy were defined as having a high mCherry/GFP fluorescence ratio. Data are presented as means of 3 independent experiments ± SD. Statistical comparison of control and treated cells is indicated (^***^P < 0.001; Two-way ANOVA test).

Since repetitive treatment had led to a reduction of intracellular ROS (Fig. 3d), we hypothesized that WAY had affected mitochondrial function and metabolism in Bjab cells. The generation of NADH and NADPH from NAD+ by dehydrogenases is critical for active metabolism and for the production of ROS. The relative content of NADH and NADPH in cells can therefore act as a proxy for the metabolic state of cells. Here, we found that 5HT1A antagonism significantly reduced dehydrogenase activity in treated Bjab cells as early as 24 h, and that this reduction was dose-dependent (Fig. 6a). Strikingly, and consistent with low intracellular ROS, we found that repeated treatment with WAY almost completely abolished dehydrogenase activity in Bjab cells at 72 h (Fig. 6b). This indicated that the antagonist might, at least to some extent, act directly on mitochondrial function.

Fluorescent probes for monitoring mitochondrial membrane potential (∆Ψm) can be used to evaluate mitochondrial function and the health status of cells^41^. We therefore measured ∆Ψm in Bjab cells at 24 h and 72 h in the presence or absence of the 5HT1A antagonist. ∆Ψm was slightly but significantly reduced in the treatment-group at 24 h (Fig. 6c) and a more prominent and significant effect was observed at 72 h, consistent with low ROS and dehydrogenase activity (Fig. 6d).

Dysfunctional mitochondria have been shown to be removed by a process of autophagy^42^. The microtubule-associated protein 1 A/1B-light chain 3 (LC3) is involved in the organelle/mitochondria sequestration and formation of autophagosomes^43,44^. Since LC3 is associated with autophagosomes from initial formation to fusion with lysosomes, LC3 detection is widely used to measure autophagy^45^. We therefore produced a Bjab cell line with constitutive expression of a tandem mCherry-EGFP-LC3B (C-G-LC3) protein, to measure autophagy in response to 5HT1A antagonism. Due to the higher sensitivity of EGFP to the acidic environment of the autolysosome relative to mCherry, it is possible to quantify autophagic flux in these cells^46^. To validate this method to quantify autophagic flux, we nutrient depleted our Bjab reporter-expressing cell line using Earle’s balanced salt solution (EBSS) in the presence and absence of Bafilomycin A1, an inhibitor of autophagic flux (Fig. 6e and Supplementary Fig. S5). Subsequently, we exposed mCherry-EGFP-LC3B-Bjab cells to single treatment or repetitive treatment of WAY for 24 h and 72 h respectively and found a significant increase in the frequency of cells with high autophagy flux at both time points (Fig. 6f).

Collectively, these data demonstrate that 5HT1A antagonism leads to rapid disruption of mitochondrial function and reduced energy production in Bjab cells. Moreover, the results indicate that damaged mitochondria may be removed by autophagy.

### Selective 5HT1A antagonism inhibits the proliferation of B cell derived lymphoma cell lines

**Figure 7:**
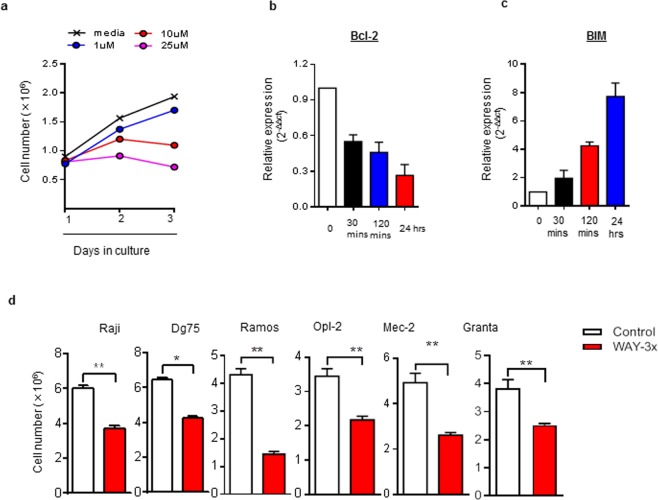
5HT1A antagonist WAY significantly inhibits the proliferation of non-BL B cell derived lymphoma cells lines. (**a**) Val, a DLBCL cell line, was treated with indicated concentrations of WAY and relative cell numbers in each culture were determined by adding a fixed number of counting beads to respective culture and calculating the ratio of Val cells to beads. (**b**,**c**) rtqPCR analysis of the Bcl-2 (anti-apoptotic) and Bim (pro-apoptotic) gene expression in Val cells exposed to 10 µM of WAY at indicated periods. Expression levels were normalized to Beta-actin, calculated by 2(−ΔΔct) method and 0 hr expression was set to 1 and expression change is represented as a fold change. (**d**) Different B cell derived lymphoma cell lines were exposed to repetitive treatments (WAY-3×; 50 µM/24 h) for 72 h and number of cells were counted at 72 h time point using TC20 Automated Cell Counter. Data is presented as the mean of 3–5 independent experiment ± SD. Statistical comparison of control and treated cells at 72 h is indicated (^**^P < 0.01; ^*^P < 0.05; Mann-Whitney test).

To determine whether 5HT1A antagonism was effective as a general tool to prevent proliferation of lymphoma cells, we treated the rapamycin resistant DLBCL cell line Val^18^ with increasing concentrations of WAY and total number of cells were counted until 72 h in culture. We found that Val cells were more sensitive to proliferation-inhibition by 5HT1A antagonism. 10 µM was sufficient enough to inhibit proliferation and treatment with 25 µM led to an almost complete block in cell proliferation (Fig. 7a). Strikingly, Val cells were extremely sensitive to WAY, an effect could be observed at the lowest tested dose (1 µM), 50 times lower than the dose used for Bjab cells (Fig. 7a). In Bjab cells, cMyc is under the control of the Ig heavy chain promoter. The Val cell line represents a “double hit” lymphoma with translocation of both cMyc and Bcl2 to the Ig heavy chain promoter^47^. Consistent with suppression of transcription via the Ig heavy chain promoter, we found decreased transcription of Bcl2 (Fig. 7b). In concert with this, we also found an up-regulation of transcripts for the apoptotic protein Bim (Fig. 7c). This suggests that 5HT1A antagonism instigate a broad inhibitory effect on the growth of lymphoma cell lines.

To further investigate this, we used repeated treatment regimen of 5HT1A antagonist WAY on multiple lymphoma cell lines to evaluate its impact on cell proliferation. After 72 h or treatment, we found that cell proliferation had been reduced in all lymphoma cell-lines tested, including the BL lines Raji, Dg75 and Ramos; the DLBCL Opl-2 the EBV + chronic lymphocytic leukemia line Mec-2 and the mantle cell lymphoma line Granta (Fig. 7d).

These data demonstrate that 5HT1A antagonism has the potential to reduce the proliferation of a large number of B cell-derived lymphoma cell lines including double hit DLBCL cell line Val.

## Discussion

In the present study, we investigated the selective antagonism of 5HT1A receptors to prevent proliferation of B cell-derived lymphoma cell lines and characterized key molecular and cellular events involved.

We demonstrated that 5HT1A antagonism blocked the proliferation of a number of B cell-derived lymphoma cell lines, including BL, DLBCL, chronic lymphocytic leukemia and mantle cell lymphoma cell lines. This blockade was associated with a transcriptional repression of genes important for cell cycle progression, metabolism and poor prognosis in cancer patients, including cMYC, GSK3-β and mutated p53^40,48,49^. Since treatment with the antagonist did not globally affect transcription or translation of all genes evaluated, our data suggested that suppression was directed at specific molecular pathways that regulate both the immunoglobulin heavy chain promoter and AKT/GSK3-β signaling pathways. The former is important due to its role in gene translocations in fast growing lymphomas and the latter is consistent with findings of 5HT1A to affect both AKT and GSK3-β phosphorylation in cultured neurons and brain^50^. The anti-proliferative effect of 5HT1A antagonism was more efficient in the rapamycin resistant cell line Val^18^. This cell line was originally derived from the bone marrow of a 50-year-old woman with B-acute lymphoblastic leukemia, that carries the three-way-translocation t(8;14;18) and overexpress Bcl2 and c-Myc, translocations that are associated with poor clinical prognosis^51,52^. It was previously shown that the strength of serotonin receptor signaling is dependent both on protein levels, but also on the presence of adaptor proteins and the strength of interaction between associated G-protein and the receptor^53^. This could explain why we did not find a direct association between 5HT1A protein levels and sensitivity to WAY treatment in Val and Bjab cells. Importantly, the effect of serotonin signaling is dependent on the presence of excitatory, or inhibitory, serotonin receptor expression. Therefore, it will be important to further characterize the expression of serotonin receptor subtypes on B cell-derived lymphomas to understand whether different receptor subtypes can predict the efficacy of 5HT1A antagonism to inhibit growth.

Growth-inhibition through antagonism of serotonin receptor signaling in fast growing tumors with these translocations would likely provide additional support to current treatment regimens to increase favorable outcome.

We found that treatment with the 5HT1A antagonist reduced the production of ROS in Bjab cells. This reduction was likely explained by a rapid induction of mitochondrial membrane depolarization together with a simultaneous increase in autophagic flux. These data suggest that a blockade of serotonin signaling via 5HT1A affect the function and integrity of mitochondria that subsequently lead to apoptosis. While it is generally accepted that autophagy functions as a mechanism to survive cellular stresses, several studies suggest that autophagy itself may be a mechanism of cell death^54^. In this case, large activations in autophagy could lead to cell death by actively degrading necessary cellular components (*e*.*g*., mitochondria)^55^. Since mitochondrial dysfunction has been shown to also modulate transcriptional responses in cells to physiological stress^56^, this suggest that the downregulation of AKT, p53 and cMyc may be an effect of prolonged 5HT1A antagonism and a sustained effect on the mitochondria.

Serotonergic signaling has been extensively studied in the context of neurotransmission and neuropharmacology to treat psychiatric disease. Taking this into consideration, a large number of well-defined and clinically approved drugs exist that may be easily evaluated and repositioned for use as an adjuvant to salvage treatment regimens for primary treatment failure of lymphoma. In particular, WAY-100,635 used in this study, was initially developed in the mid 1990’s as a specific radioligand to visualize 5HT1A in the brain using PET and today is still used in experimental brain imaging^57,58^. The specific pharmacokinetics and pharmacodynamics of WAY-100,635 have been extensively studied in addition to modifications of this compound with limited ability to cross the blood brain barrier^59^. Peripheral 5HT1A antagonism may be more suited to treat lymphoma without CNS involvement to avoid any possible central side effects. On the contrary, brain penetrant WAY-100,635 could prove highly valuable in lymphoma patients with CNS involvement where treatment options are further limited. Blood-brain barrier disruption has been primarily used to treat CNS lymphoma, although the superiority of this technique over conventional treatment is still debated^60^.

Unique and innovative novel strategies are urgently needed to address lymphoma with primary treatment failure. Collectively, we demonstrate the potential of a selective 5HT1A antagonist for treatment of B cell derived lymphomas. Further investigations to understand whether our results translate to *in vivo* inhibition of B cell lymphoma growth are highly warranted.

## Supplementary information

Growth-inhibition of cell lines derived from B cell lymphomas through antagonism of serotonin receptor signaling.

## Data Availability

All relevant data to support the findings within this study are available upon request from the corresponding author.
