# Supplementary material for: Growth-inhibition of cell lines derived from B cell lymphomas through antagonism of serotonin receptor signaling
Source: Sci Rep. 2019 Mar 12;9:4276. doi: 10.1038/s41598-019-40825-x (PMC6414675; doi:10.1038/s41598-019-40825-x)
Supplement: Supplementary file 1 — Growth-inhibition of cell lines derived from B cell lymphomas through antagonism of serotonin receptor signaling. [file 41598_2019_40825_MOESM1_ESM.pdf]

Growth-inhibition of cell lines derived from B cell lymphomas through antagonism of serotonin receptor signaling.

Shrikant S Kolan[1], Tommy Lidström[1], Tomás Mediavilla[2], Andy Dernstedt[1], Sofie Degerman[3], Magnus Hultdin[3], Karl Björk[1], Daniel Marcellino[2] and Mattias N.E. Forsell[1].

[1] Department of Clinical Microbiology, Section of Infection and Immunology, Umeå University, Umeå, Sweden.

[2] Department of Integrative Medical Biology, Umeå University, Umeå, Sweden.

[3] Department of Medical Biosciences, Umeå University, Umeå, Sweden.

This supplementary file includes:

1. Online extended methods
2. Supplementary Figure 1
3. Supplementary Figure 2
4. Supplementary Figure 3
5. Supplementary Figure 4
6. Supplementary Figure 5

[1] Correspondence: Mattias N. E. Forsell (mattias.forsell@umu.se)

Supplementary Information:

Online extended methods

Reagents

The following Taqman gene expression assays (Applied Biosystems) containing pre-designed primers and probes were used in rtqPCR experiments: 5HT1A (Hs00265014_s1); cMYC (Hs00153408_m1); p53 (Hs01034249_m1); GSK3-β (Hs01047719_m1); AKT1 (Hs00178289_m1); Beta-actin (Hs99999903_s1), 18s rRNA (Hs99999901_s1), Bcl2 (Hs00608023_m1) and Bcl2L11 (BIM) (Hs_00236329_m1). For western blot experiments primary antibodies against cMYC (#5605S)[1], TP53 (#2527S)[2], pAKT-s473 (#9271S)[3], Total AKT (#9272S)[4], pGSK3B (#9323S)[5], Total GSK3B (#9315S)[6], Beta-actin (#4967S)[7] were purchased from Cell Signaling. Anti-pH2AX-ser139 (05-636)[8] antibody was from Millipore, Cyclophilin A from Thermo Fischer (#PA1-025)[9] whereas primary rabbit polyclonal anti-5-HT1A antibody was purchased from Santa Cruz Biotechnology (Santa Cruz, CA; catalog number sc 10801)[10]. In flow cytometry experiment anti-humanCD19 antibody (#562321) and fixable viability dye 780 (#565388) from was purchased from BD biosciences.

Apoptosis and ds-DNA damage analysis

Analyses for apoptosis was measured by using Annexin-V FITC/PI apoptosis detection kit (BD Biosciences). In brief, control and WAY treated Bjab cells were harvested at 72h, washed two times with PBS and resuspended in 1x binding buffer at a concentration of $1x10^6$ cells/ml. Next, $1x10^5$ cells in 100µl were transferred into 5ml tubes and then stained with PI (5µl) and Annexin-V fluorescein isothiocynate (FITC; 5µl) FITC for 15 mins at RT in the dark. Finally, 400µl of 1x binding buffer was added to each tube and samples were analyzed within 1h using LSRII (BD) flow cytometer with FlowJo v10.0.7 (FlowJo, LLC) software.

At 72h control or WAY treated Bjab cells were harvested, washed with PBS and suspended in 100µl per tube of cytofix/ cytoperm fixation and permeabilization solution and incubated for 15-30 mins on ice. Next, cells were washed with 1x Perm/Wash buffer and incubated in 20µl of perm/wash buffer containing anti-H2AX (ps139; 5µl) and anti-cleaved PARP (Asp214; 5µl) antibody, for 20 minutes at RT. Finally, cells were washed and analyzed using LSRII (BD) flow cytometer with FlowJo v10.0.7 (FlowJo, LLC) software.

Measurement of Reactive oxygen species (ROS):

Intracellular ROS formation was detected using the dye 2′, 7′ -dichlorodihydrofluorescein diacetate (H2DCFDA-Thermo Fischer) as per manufactures protocol. At 72h control or WAY treated Bjab cells were harvested, washed with PBS and incubated with H2DCFDA dye (10µM) at 37 °C for 30 mins. After incubation, cells were washed with warm PBS and fluorescence intensity was measured by flow cytometry (Accuri- BD Biosciences) and data was analyzed using FlowJo v10.0.7 (FlowJo, LLC) software.

Mitochondrial membrane potential (ΔΨm) analysis

Mitochondrial membrane potential (ΔΨm) was detected by flow cytometry using JC-1 staining following the manufacturer protocol (JC-1 Mitochondrial Membrane Potential Detection Kit, Thermo Fischer). Untreated and WAY treated Bjab cells

were harvested at 24 or 72h, washed twice with warm HBSS followed by the addition of 500µl of JC-1 (5µM) staining solution. Further samples were incubated at 37°C for 15 mins in a 5% CO_2 incubator. Finally, stained cells were washed two times with warm HBSS and immediately analyzed using LSRII (BD Biosciences) flow cytometer with FlowJo v10.0.7 (FlowJo, LLC) software.

Protein expression

For Western blot, proteins were extracted and lysed in RIPA buffer containing protease and phosphatase inhibitors (Thermo Fischer). Thirty micrograms of protein was electrophoretically separated on 4–12% NuPAGEo Bis-Tris Gels (Life technologies) and then transferred onto nitrocellulose membrane. To block non-specific binding, membranes were incubated with 5% bovine serum albumin (BSA) diluted in TBST (0.05% tween-20 in PBS) for 1h. Further, membranes were probed with primary antibodies against cMYC, p53, pAKT-s473, Total AKT, pGSK3β-s9, total GSK3-β, Beta-actin, pH2AX-ser139, 5HT1AR and Cyclophilin A, diluted in TBST with 5% BSA overnight at 4 degree. Next day, membrane was washed and incubated with HRP-conjugated goat-anti rabbit IgG (1:5000) for 1h RT. The protein signal was developed using Supersignal west pico chemiluminescent substrate (Thermo Fischer) and visualized using imageQuant LAS4000 (GE Healthcare), band intensity was quantified using image analysis software from Li-Cor (Lincoln).

Determination of autophagy using flow cytometry

- **Preparation of mCherry-EGFP-hLC3 retroviral particles**

The pBabe-mCherry-GFP-LC3 γ-retroviral transfer plasmid was used to generate mCGhLC3 retroviral particles. 293T/17 cells (ATCC CRL-11268) were transfected with a combination (ratio: 1/0.8/0.1) of the pBabe-mCherry-GFP-hLC3, pCMV-VSV-G and pUMVC plasmids using polyethylenimine (Polysciences, Inc.) to produce murine leukemia virus (MuLV) retroviral particles collected at 24 and 48h after infection from the cell culture supernatant. pBABE-puro mCherry-EGFP-LC3B was a gift from Jayanta Debnath (Addgene plasmid 22418), whereas pCMV-VSV-G and pUMVC were a gift from Bob Weinberg (Addgene plasmid 8454 and 8449, respectively).

- **Generation of the mCherry-EGFP-hLC3 reporter cell line (Bjab-mCGhLC3)**

Bjab cells were infected with retroviral particles carrying pBabe puro-mCherry-GFP-hLC3 in the presence of 80 ng/mL polybrene (Merck Millipore) and subsequently selected with puromycin (1 µg/ml) to obtain a stable cell line. Stable Bjab-mCGhLC3 cells were sorted for fluorescence uniformity using a BD FACSAria™ III (BD Biosciences) and a subpopulation of cells expressing high levels of the tandem mCherry-EGFP reporter were separated for their expansion and later experimental use.

References:

1 Shavlakadze, T. *et al.* Short-term Low-Dose mTORC1 Inhibition in Aged Rats Counter-Regulates Age-Related Gene Changes and Blocks Age-Related Kidney Pathology. *J Gerontol A Biol Sci Med Sci* **73**, 845-852, doi:10.1093/gerona/glx249 (2018).

2 Yang, L., Li, Y., Bhattacharya, A. & Zhang, Y. PEPD is a pivotal regulator of p53 tumor suppressor. *Nature Communications* **8**, 2052, doi:10.1038/s41467-017-02097-9 (2017).

3 Mishall, K. M. *et al.* Sustained activation of the AKT/mTOR and MAP kinase pathways mediate resistance to the Src inhibitor, dasatinib, in thyroid cancer. *Oncotarget* **8**, 103014-103031, doi:10.18632/oncotarget.20488 (2017).
4 Lin, Y. *et al.* PIK3R1 negatively regulates the epithelial-mesenchymal transition and stem-like phenotype of renal cancer cells through the AKT/GSK3beta/CTNNB1 signaling pathway. *Sci Rep* **5**, 8997, doi:10.1038/srep08997 (2015).
5 Hou, T. *et al.* CLCA4 inhibits bladder cancer cell proliferation, migration, and invasion by suppressing the PI3K/AKT pathway. *Oncotarget* **8**, 93001-93013, doi:10.18632/oncotarget.21724 (2017).
6 Lee, M. S. *et al.* PI3K/AKT activation induces PTEN ubiquitination and destabilization accelerating tumourigenesis. *Nat Commun* **6**, 7769, doi:10.1038/ncomms8769 (2015).
7 Zou, Z. *et al.* Aurora kinase A inhibition-induced autophagy triggers drug resistance in breast cancer cells. *Autophagy* **8**, 1798-1810, doi:10.4161/auto.22110 (2012).
8 Smith-Roe, S. L. *et al.* SWI/SNF complexes are required for full activation of the DNA-damage response. *Oncotarget* **6**, 732-745, doi:10.18632/oncotarget.2715 (2015).
9 Bartz, R. *et al.* Evidence that mono-ADP-ribosylation of CtBP1/BARS regulates lipid storage. *Mol Biol Cell* **18**, 3015-3025, doi:10.1091/mbc.e06-09-0869 (2007).
10 Ou, X. M. *et al.* Freud-1: A neuronal calcium-regulated repressor of the 5-HT1A receptor gene. *J Neurosci* **23**, 7415-7425 (2003).

Supplementary Figure 1

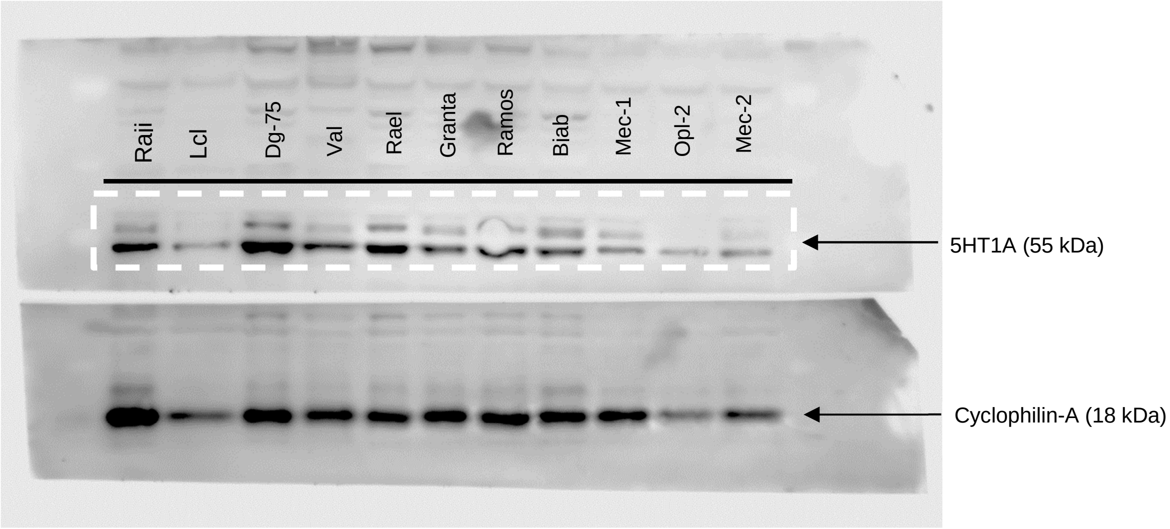

Supplementary Figure 1. Full scanned gels for western blot shown in figure 1C. White dashed line identifies cropped region shown in respective figure. Cyclophilin-A was used as a loading control. Assays were repeated at least three times and representative blots are shown.

Supplementary Figure 2

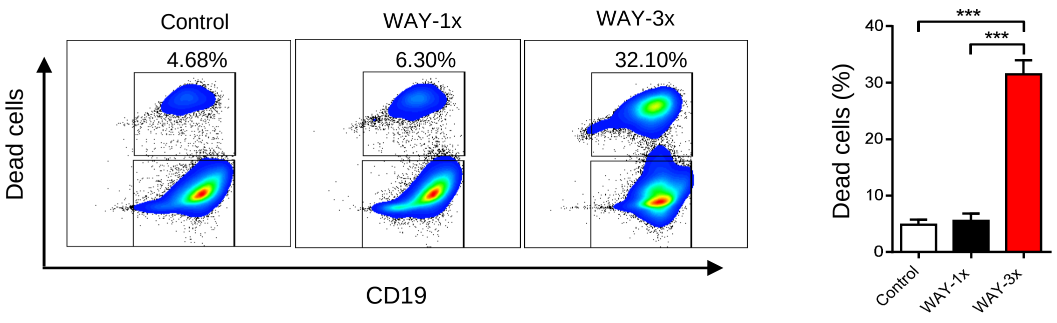

Supplementary Figure 2. Repetitive treatments of the 5HT1A antagonist WAY results in disrupted membrane integrity in Bjab cells. **(A)** Representative flow cytometric analysis plots for the determination of cells disrupted membrane integrity at 72h. Bjab cells were exposed to single (WAY-1x; 50µM) and repetitive treatments (WAY-3x; 50µM/24h) for 72h, stained with fixable viability dye and CD19, and analyzed by flow cytometry. Data is presented as the mean of 3 independent experiments ± SD. Statistical comparison of control and treated cells is indicated (*** $P<0.001$; Two-way ANOVA test).

Supplementary Figure 3

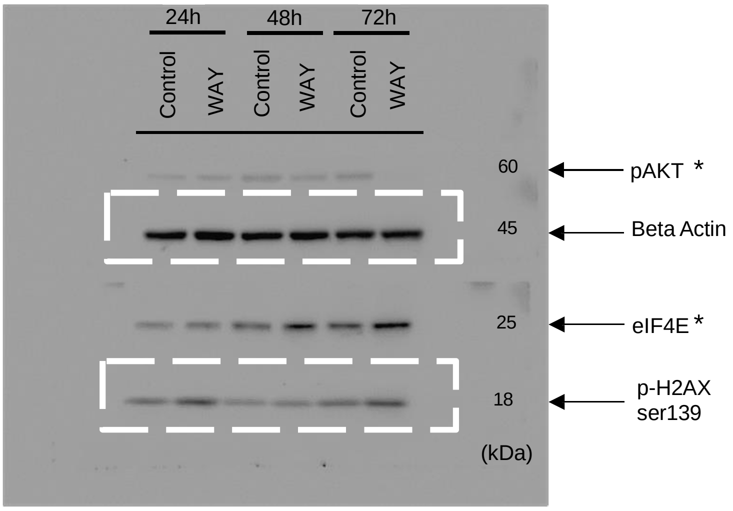

Supplementary Figure 3. Full scanned gels for western blot shown in figure 3B. White dashed line identifies cropped region shown in respective figure. Some part of the gel image was cropped to remove samples unrelated to study which were ran on the same gel. Samples marked with *(pAKT and eIF4E) were not included in this experiment. Beta actin was used as a loading control, assays were repeated at least three times and representative blots are shown.

Supplementary Figure 4

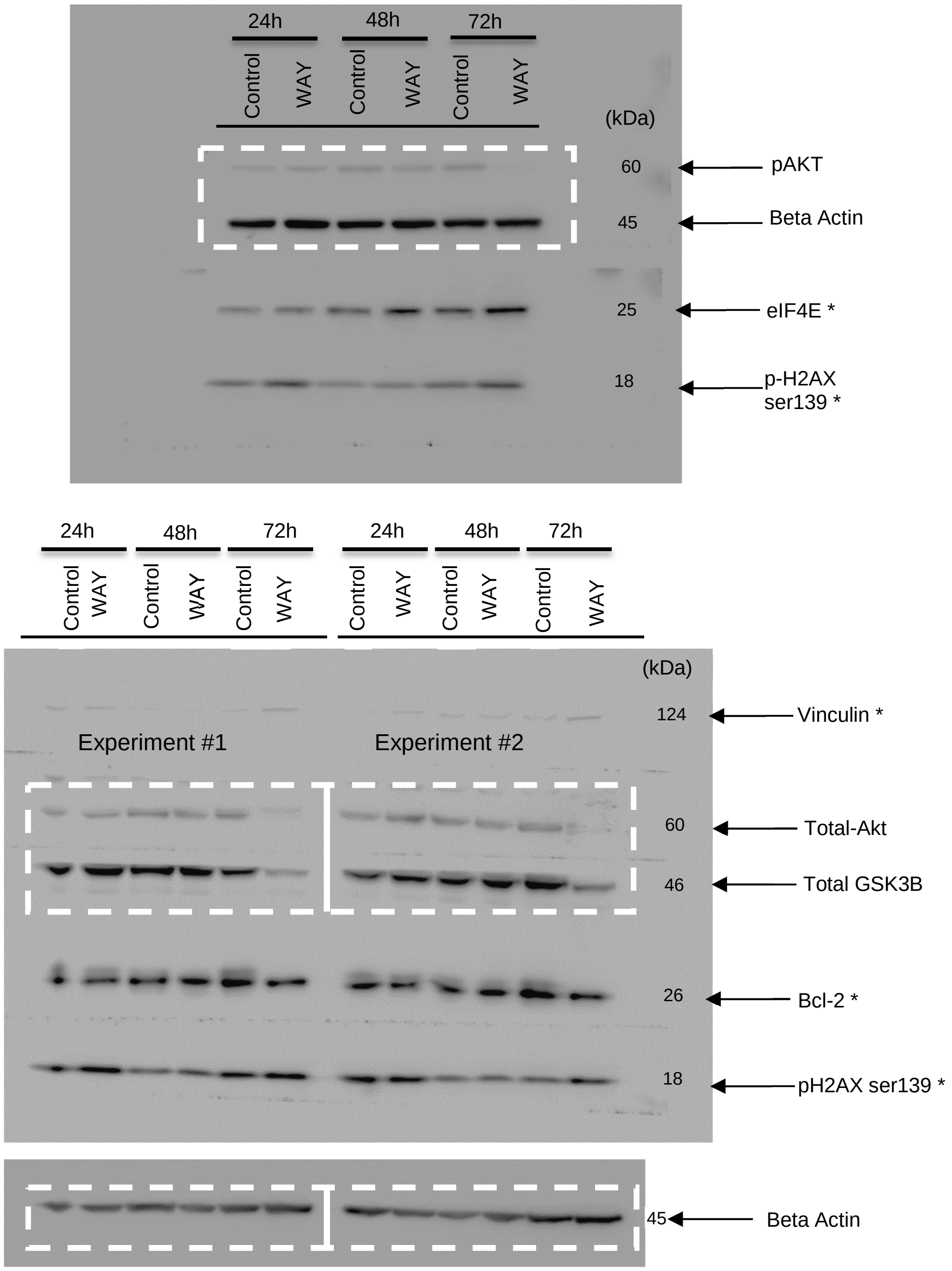

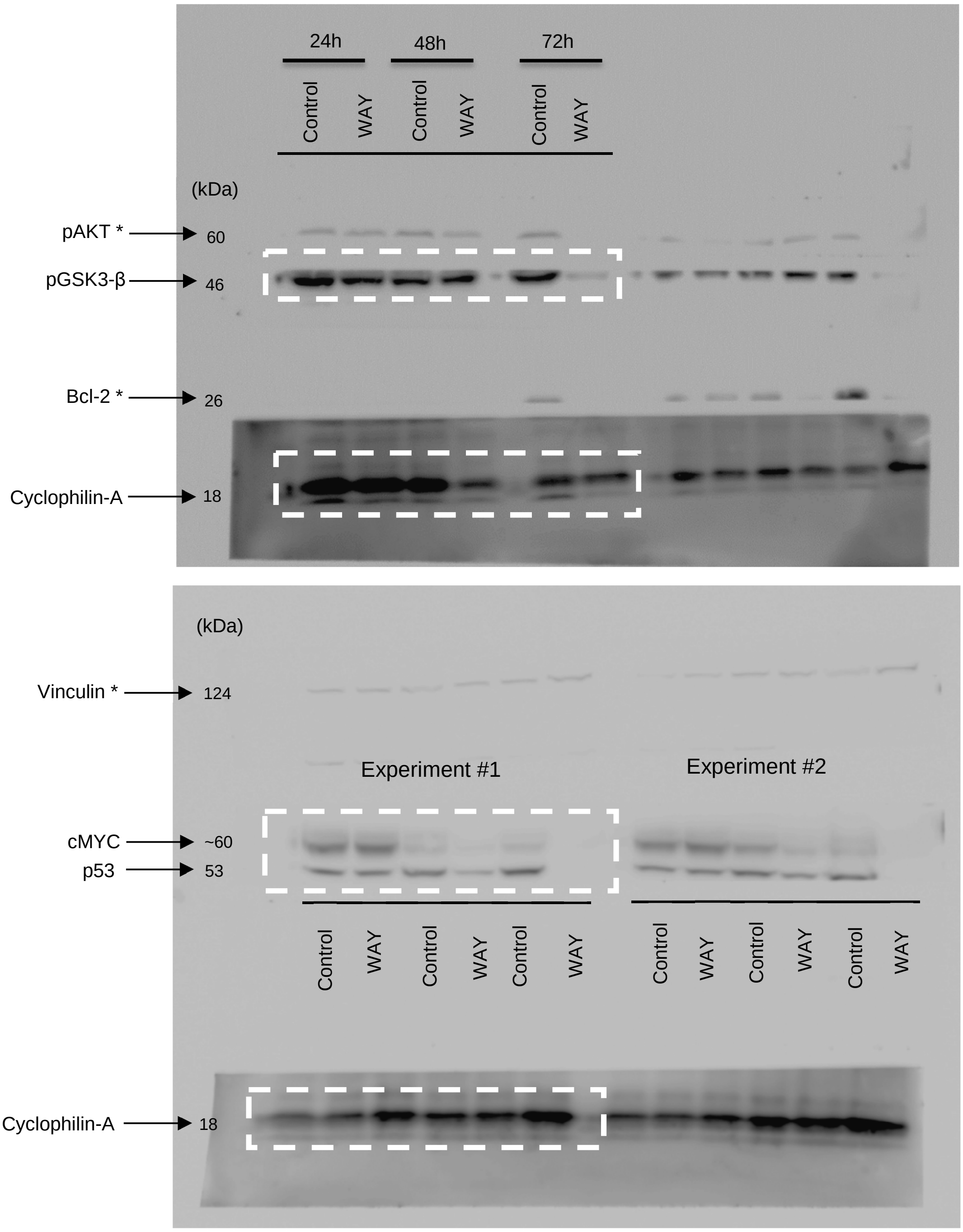

Supplementary Figure 4. Full scanned gels for western blot shown in figure 5C. White dashed line identifies cropped region shown in respective figure. Some part of the gel image was cropped to remove samples unrelated to study which were ran on the same gel. Samples marked with * were not included in the respective experiments. Beta actin or Cyclophilin-A was used as a loading control as mentioned in respective blots. Assays were repeated at least three times and representative blots are shown.

Supplementary Figure 5

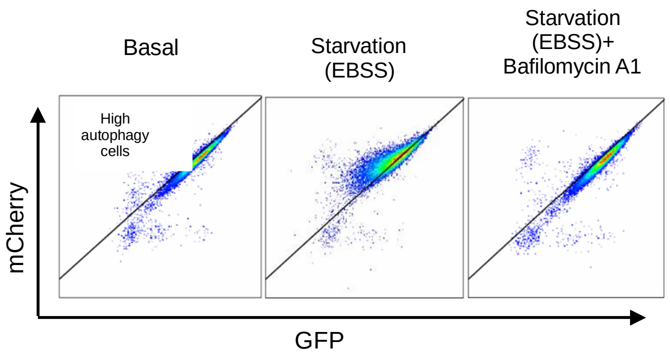

Supplementary Figure 5. Validation of autophagy experiment. (**A**) Representative flow cytometric analysis for mCherry-EGFP-LC3B-expressing Bjab cells treated with Earle's BalSalt Solution (starvation is the most potent autophagy stimulus) and Earle's Balanced Salt Solution (EBSS) in the presence of Bafilomycin A1 (autophagy inhibitor).
